# When will the battle against novel coronavirus end in Wuhan: A SEIR modeling analysis

**DOI:** 10.7189/jogh.10.011002

**Published:** 2020-06

**Authors:** Kangkang Wan, Jing Chen, Changming Lu, Lanlan Dong, Zhicheng Wu, Lianglu Zhang

**Affiliations:** 1 Wuhan Ammunition Life-Tech Co Ltd, Wuhan, Hubei, China,; 2 Clinics of Oilcrops Research Institute, CAAS, Wuhan, Hubei, China

## Abstract

**Background:**

Recent outbreak of 2019-nCoV in Wuhan raised serious public health concerns. By February 15, 2020 in Wuhan, the total number of confirmed infection cases has reached 37 914, and the number of deaths has reached 1123, accounting for 56.9% of the total confirmed cases and 73.7% of the total deaths in China. People are eager to know when the epidemic will be completely controlled and when people's work and life will be on the right track.

**Method:**

In this study we analyzed the epidemic dynamics and trend of 2019-nCoV in Wuhan by using the data after the closure of Wuhan city till February 12, 2020 based on the SEIR modeling method.

**Results:**

The optimal parameters were estimated as R_0_ = 1.44 (interquartile range: 1.40-1.47), TI = 14 (interquartile range = 14-14) and TE = 3.0 (interquartile range = 2.8-3.1). Based on these parameters, the number of infected individuals in Wuhan city may reach the peak around February 19 at about 47 000 people. Once entering March, the epidemic would gradually decline, and end around the late March. It is worth noting that the above prediction is based on the assumption that the number of susceptible population N = 200 000 will not increase. If the epidemic situation is not properly controlled, the peak of infected number can be further increased and the peak time will be a little postponed. It was expected that the epidemic would subside in early March, and disappear gradually towards the late March.

**Conclusions:**

The epidemic situation of 2019-nCoV in Wuhan was effectively controlled after the closure of the city, and the disease transmission index also decreased significantly. It is expected that the peak of epidemic situation would be reached in late February and end in March.

Wuhan is the largest city in central China with a total population of more than 11 million [1]. The epidemic of 2019-nCoV pneumonia has been raging in the whole country, especially in Hubei province for nearly a month. In late December 2019, 67 cases of 2019-nCoV pneumonia were reported in Wuhan [2]. In order to prevent the further spread of 2019-nCoV, Wuhan began to close the city from 10:00 on January 23, banning all vehicles from entering and leaving the city. Tens of thousands of medical staff, soldiers and people from all walks of life have been involved in the campaign.

The spread of the epidemic has caused a huge threat to people's health and life safety, at the same time, it has a serious impact on China's social life and national economy. By February 15, 2020, the total number of confirmed cases has reached 37 914, and the number of deaths has reached 1123 in Wuhan, accounting for 56.9% of the total confirmed cases and 73.7% of the total deaths in China [3]. With the increase of medical staff from all over the country, the opening of several large novel hospitals, and the adoption of anti epidemic measures, more patients can get efficient and timely treatment. The number of confirmed cases increased sharply on February 12 and 13, while the total number of suspected cases decreased gradually [3].

People are eager to know when the epidemic will be completely controlled and when people's work and life will be on the right track. In order to help the public to understand the future trend of the epidemic, we analyzed the epidemic dynamic and trend of 2019-nCoV in Wuhan city by using the SEIR modeling method based on the actual data and published references.

## METHODS

### Epidemic transmission model

**Figure 1:**
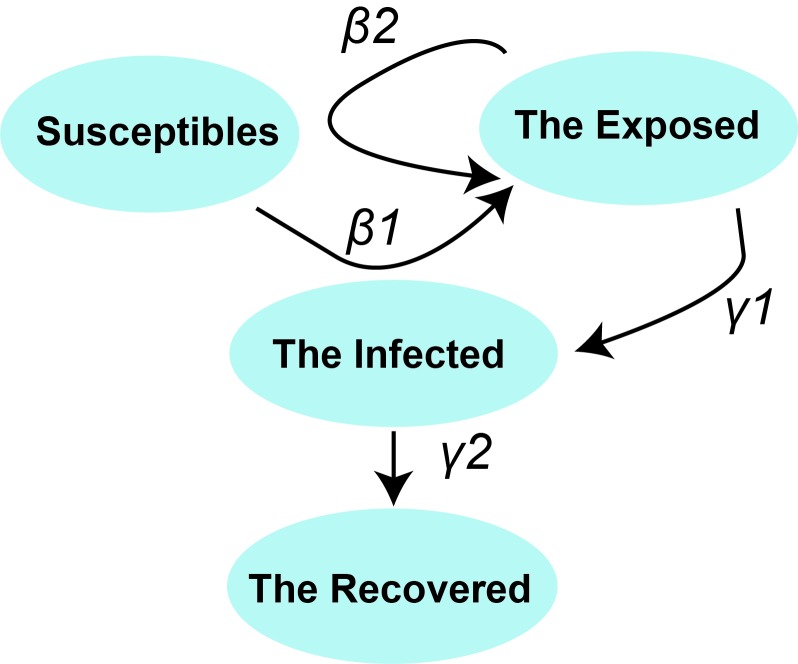
Relationship among four groups according to SEIR model.

The SEIR model is a classical epidemic model for the flows of people between four states: susceptible (S), exposed (E), infected (I), and recovery (R). Each of those variables represents the number of people in those groups. The relationship among the four groups is elucidated in **Figure 1**, where *β1* is the probability of S to E after I contacts S, *γ1* is the probability of E to I, and *γ2* is the probability of I to R. Since 2019-nCoV is also infectious in the incubation period, we introduced parameter *β2* here to represent the probability of S to E after E contact S. We used the “susceptible – exposed – infected – recovered” model [4] to describe the prevalent characteristics of 2019-nCoV in Wuhan.

This is an ordinary differential equation model, described by the following equations:

dS_(t)_/d_(t)_ = –β_1_ × I_(t)_ × (S_(t)_/N) – β2 × E_(t)_ × (S_(t)_/N)

dE_(t)_/d_(t)_ = β_1_ × I_(t)_ × (S_(t)_/N) + β2 × E_(t)_ × (S_(t)_/N) – γ_1_ × E_(t)_ 

dI_(t)_/d_(t)_ =  γ_1_ × E_(t)_ – γ_2_ × I_(t)_ 

dR_(t)_/d_(t)_ =  γ_2_ × I_(t)_ 

Among which, *S(t)*, *E(t)*, *I(t)* and *R(t)* represent the number of people in the group of the susceptible, the exposed, the infected and the recovered on the day t, respectively. N is the total number of possible contact people, which is assumed to be fixed and N = S + E + I + R.

### Estimation of parameters for the model

Parameters *β1*, *β2*, *γ1* and *γ2* were estimated according to the reference [4] using the formula below:

β_1_ = R_0_/TI

β_2_ = R_0_/TE

γ_1_ = 1/TE

γ_1_ = 1/TI

among which, R_0_ is the basic reproduction number, TI is the time of infectious period, TE is the time of incubation period, and *β_1_*, *β_2_*, γ_1_ and *γ_2_* share the same meanings as in **Figure 1**.

The optimal TE, TI, and R_0_ values were estimated by setting TE at the range of 1-7, TI at 1-14, and R0 at 1-5 according to the [5,6]. For TI and TE, the values were taken with step = 0.1 in their respective intervals. For R_0_, the values were taken with step = 0.01 from 1 to 5. Since there are three parameters in the model, we defined the value of TE, TI and R_0_ as a parameter combination. The number of individuals infected (Î) and recovered (Ȓ) for each parameter combination was calculated by substituting the values of these three parameters into the SEIR model. The root mean squared error (RMSE) of each parameter combination was calculated by following formula:

RMSE_(I)_ = sqrt[1/n ×(Î – I)]

RMSE_(R)_ = sqrt[1/n ×(Ȓ – R)]

where Î and Ȓ are estimated number of the infected and the recovered, I and R are real number of the infected and the recovered we collected. For all combinations of these three parameters, we selected the parameters which had the smallest value of RMSE_(I)_ + RMSE _(R)_ as the optimal TE, TI, and R_0_. In order to avoid model over-fitting with this method, we randomly sampled 80% of the data for fitting each time, and repeated this for 100 times, and finally we used the average of the fitted TI, TE and R_0_ for 100 times as the model's optimal TI, TE and R_0_.

### Data source

The data were collected from the official website of Hubei Provincial Health Committee (http://wjw.hubei.gov.cn/) [3], and shown in **Table 1**. We used the data of 22 days from January 22 to February 12 when Wuhan city was shut down and all the public transportation was suspended.

**Table 1:** Number of the infected and the recovery at specific date in Wuhan*

Date (2020)	Infected	Recovered
22 Jan	425	28
23 Jan	495	31
24 Jan	572	39
25 Jan	618	40
26 Jan	698	42
27 Jan	1590	45
28 Jan	1905	75
29 Jan	2261	82
30 Jan	2639	103
31 Jan	3215	139
1 Feb	4109	171
2 Feb	5142	224
3 Feb	6384	303
4 Feb	8351	368
5 Feb	10 117	431
6 Feb	11 618	534
7 Feb	13 603	698
8 Feb	14 982	877
9 Feb	16 902	1044
10 Feb	18 454	1206
11 Feb	19 588	1377
12 Feb	32 944	1915

*Source: http://wjw.hubei.gov.cn/

For construction of the model, data of 22 days were divided into two stages. The first stage is from January 23 to February 7, and the second stage is from February 8 to February 12. During the second stage, Wuhan city took a number of measures, including timely diagnosis, timely treatment and effective isolation of the infected population, which will have an important impact on the parameters of the model.

### Initial parameter settings

To establish the model, we first estimated the parameters of the susceptible (S), the exposed (E), the infected (I) and the recovery(R) based on the latest data available on February 12:

N = 200 000, which is the total number of potential close contacts in Wuhan on February 12.

S = N – I, in which S is the number of the susceptible and I is the number of the infected.

I_(0)_ = 425, which is the number of susceptible individuals at the beginning of the model run.

E_(0)_ = 426, which is the number of exposed individuals at the beginning of the model run

R_(0)_ = 28, which is the number of recovered individuals at the beginning of the model run.

## RESULTS

### Epidemic prediction based on SEIR model

The epidemic of the novel coronavirus pneumonia in Wuhan was studied by SEIR modeling. The results showed that, at the time when Wuhan was closed, the number of initially infected individuals was I_(0)_ = 425, the number of initially exposed individuals was E_(0)_ = 426, and the number of initially recovered patients was R_(0)_ = 28.

**Figure 2:**
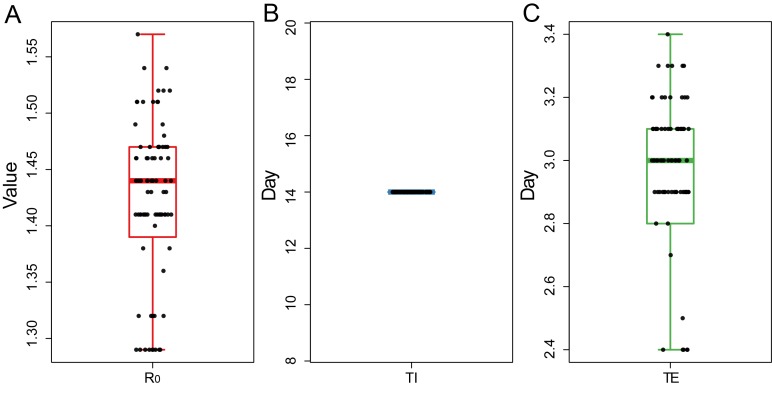
Distribution of the R0 (**Panel A**), TI (**Panel B**) and TE (**Panel C**) for 100 random samplings.

Next, we separated the data into two stages: January 22-February 7 and February 8-February 12. In the first stage, TI = 14 (interquartile range = 14-14), TE = 3.0 (interquartile range = 2.8-3.1), R_0_ = 1.44 (interquartile range = 1.40-1.47) (**Figure 2****,** Appendix S1 in the **Online Supplementary Document**). The data showed that the infectious time of the infected person (I) is 14 days, and the incubation period is about 3 days, which is close to the data (mean TI = 6.4 days, min-max = 0-24 days) estimated in the reference [7,8]. The propagation base R_0_ of this study is 1.44, which is significantly lower than the R_0_ estimated by other papers before the closure of Wuhan [9-11].

**Figure 3:**
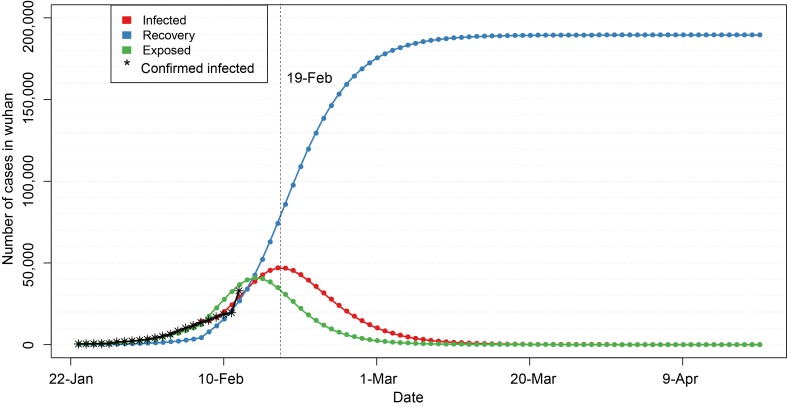
Epidemic trend of 2019-nCoV within 90 days after the closure of Wuhan.

In the second stage (after February 8), we set the number of susceptible population N to be fixed at 200 000, and the infection cycle of infected population decreased from 14 days to 4 days, ie, TI = 4, which was estimated according to the data of 5 days from February 8 to February 12, so as to get the epidemic development trend of 90 days since January 22, including the number of infected people, the number of latent people and the number of recovered people (**Figure 3****,** Appendix S2 in the **Online Supplementary Document**). The results showed that the number of infected people increased slowly in the early stage (January 22 – January 31), but during February 1 – February 12, the number of infected people increased rapidly, which is expected to peak around February 19, reaching about 47 000 people. Subsequently, the number of infections will decrease. Once entering March, the epidemic would gradually decline, and the epidemic would end around the end of March. It is worth noting that the above prediction is based on the assumption that the number of susceptible population N = 200 000 will not increase.

**Figure 4:**
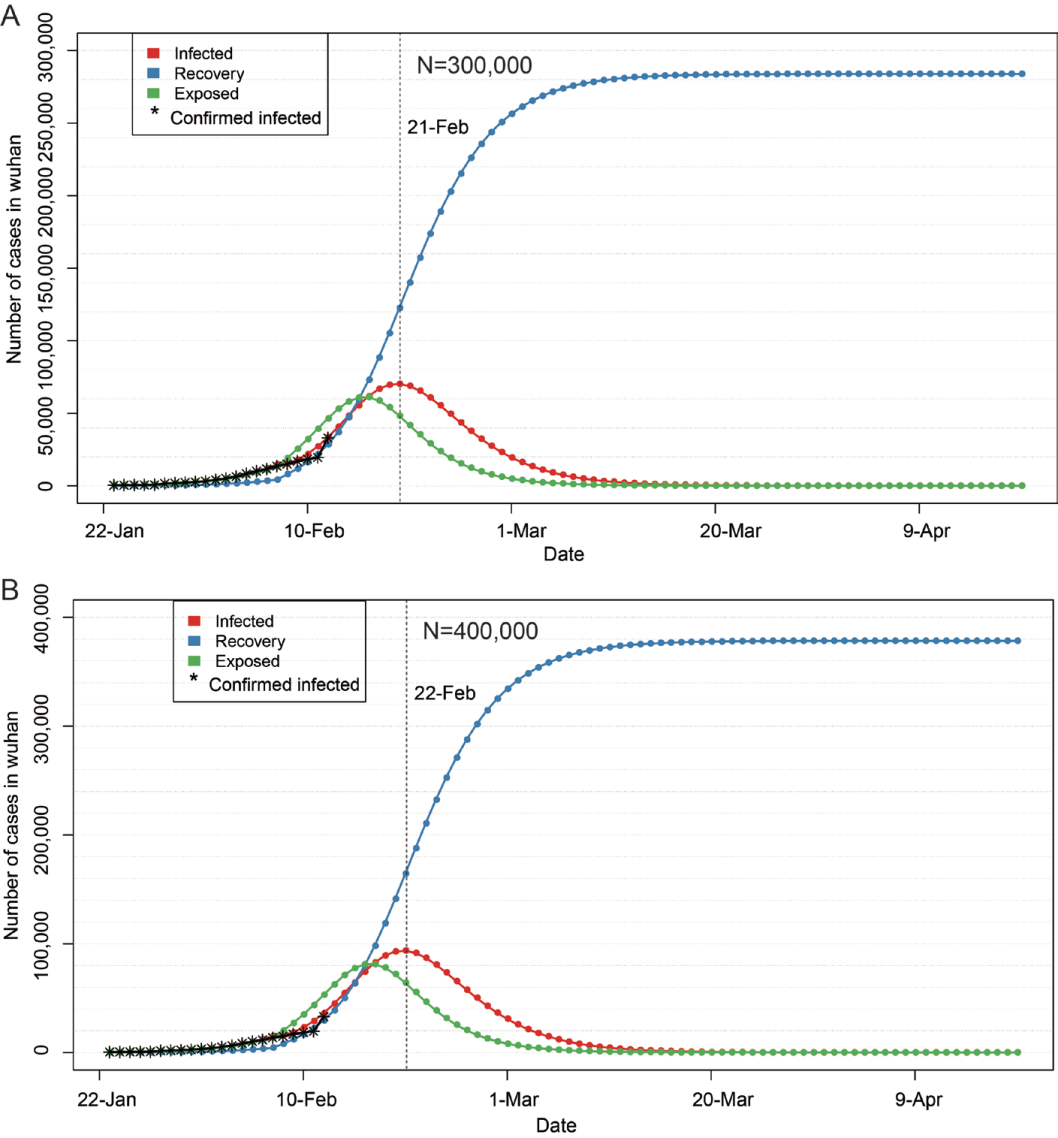
Epidemic trend of 2019-nCoV within 90 days after the closure of Wuhan city. **Panel A.** Assuming the number of susceptible people n = 300000. **Panel B.** Assuming the number of suspectible people n = 400000.

In **Figure 3** and **Figure 4**, red line indicates the trend of cumulative infection number over time, blue line is the trend of cumulative rehabilitation number over time, and green line is the trend of cumulative latent number over time. Vertical dash line indicates the peak time of cumulative infection number.

If the epidemic situation is not properly controlled, the number of susceptible population will continue to increase on the basis of current N = 200 000. If the number of susceptible population increases to N = 300 000, and other parameters remain unchanged, the peak number can increase to 75000, and the epidemic peak time will also be postponed at around February 21 (**Figure 4**). If it is increased to N = 400 000 and other parameters remain unchanged, the peak number can be increased to 100 000, and the epidemic peak will be postponed to around February 22 (**Figure 4**). Even in both cases, the epidemic would subside in early March, and disappear gradually towards the late March.

## DISCUSSION

Although some modeling studies on the epidemiological characteristics of 2019-nCoV epidemic have been reported so far, they had some limitations, such as the data come from the early stage of the epidemic. Due to the rapid change of the epidemic situation and the closure of Wuhan on January 23, many parameters related to the model have also changed, which affect the applicability and reliability of the model. This study used the latest 2019 nCoV data in Wuhan area, analyzed the epidemiological characteristics of 2019 nCoV epidemic after Wuhan city was shut down. Compared with other studies, the R_0_ value produced in this study is smaller, indicating that the closure and subsequent measures have played an important role in the spread of the epidemic.

The infection time index (TI) obtained in this study was higher than that of SARS [12] and MERS [13], but lower than that of 2019-nCoV in literatures [14] reported earlier. This result may be related to the sudden outbreak of the epidemic, the lack of medical resources for early response, and the failure of timely diagnosis and treatment of infected patients. A large number of mild patients and asymptomatic virus carriers were not isolated in time. The incubation period (TE) is about 3 days, which is close to the data in the reference [14].

According to the latest reported data, the cumulative number of people infected on February 13 and 14 was 35 991 and 37 914 respectively, which is close to the number predicted by our estimation (Appendix 2 in the **Online Supplementary Document**). According to this study, the number of infected people will reach the peak in February 19 at about 47 000 infected individuals. It should be noted that the development of the epidemic is rapid, especially with the external factors involved, the model-related parameters are also dynamically changing. Therefore, with the latest data being added, the values of R_0_, TI, and TE will also be changed. It is foreseen that both R_0_ and TI will further decline, which means that breakthroughs in the epidemic should be gradually arrived.

## CONCLUSIONS

With the implementation of more follow-up measures, including strict restrictions on people going out, accelerating the treatment of infected individuals, and clinical trials of new drugs, the development of 2019-nCoV epidemic in Wuhan will be effectively controlled, and the number of infected individuals will gradually decrease. It was expected that the epidemic would subside in early March, and disappear gradually towards the late March. If the epidemic situation is not properly controlled, the peak of infected number can be further increased and the peak time will be a little postponed.

## Additional material

Online Supplementary Document
