# Supplementary material for: When will the battle against novel coronavirus end in Wuhan: A SEIR modeling analysis
Source: J Glob Health. 2020 Apr 1;10(1):011002. doi: 10.7189/jogh.10.011002 (PMC7125416; doi:10.7189/jogh.10.011002)
Supplement: Online Supplementary Document [file jogh-10-011002-s001.pdf]

Appendix S1. Distribution of the R_0 (A), TI (B) and TE (C) for 100 random samplings

Repeats	R_0	TI	TE	$RMSE_{(I)}$	$RMSE_{(R)}$	$RMSE_{(I)}$ +$RMSE_{(R)}$
1	1.54	14.00	3.30	232.06	1108.30	1340.36
2	1.29	14.00	2.30	328.27	1514.78	1843.05
3	1.29	14.00	2.30	278.94	1446.31	1725.26
4	1.41	14.00	2.90	325.89	1335.01	1660.90
5	1.51	14.00	3.20	259.15	1237.79	1496.94
6	1.57	14.00	3.40	235.95	1115.25	1351.20
7	1.44	14.00	3.00	336.74	1549.45	1886.20
8	1.54	14.00	3.30	276.54	1203.22	1479.76
9	1.36	14.00	2.50	230.20	1110.36	1340.56
10	1.44	14.00	3.00	328.24	1525.51	1853.75
11	1.44	14.00	3.00	333.88	1510.17	1844.05
12	1.26	14.00	2.20	239.48	1420.70	1660.18
13	1.47	14.00	3.10	344.07	1492.23	1836.30
14	1.41	14.00	2.90	324.17	1307.48	1631.65
15	1.41	14.00	2.90	265.93	1492.69	1758.62
16	1.29	14.00	2.30	329.13	1524.16	1853.29
17	1.46	14.00	3.10	307.72	1181.57	1489.29
18	1.41	14.00	2.90	273.56	1492.94	1766.51
19	1.51	14.00	3.20	257.96	1180.79	1438.75
20	1.26	14.00	2.20	258.77	1409.80	1668.56
21	1.46	14.00	3.10	312.57	1389.26	1701.83
22	1.46	14.00	3.10	319.22	1223.58	1542.81
23	1.43	14.00	3.00	294.43	1188.60	1483.03
24	1.44	14.00	3.00	330.24	1507.94	1838.18
25	1.69	14.00	3.70	199.45	955.89	1155.34
26	1.41	14.00	2.90	324.01	1293.32	1617.33
27	1.46	14.00	3.10	314.87	1391.49	1706.36
28	1.29	14.00	2.30	316.01	1523.79	1839.79
29	1.47	14.00	3.10	343.78	1523.28	1867.06
30	1.51	14.00	3.20	264.95	1224.86	1489.81
31	1.52	14.00	3.30	325.20	1370.43	1695.63
32	1.43	14.00	3.00	202.23	1271.15	1473.37
33	1.44	14.00	3.00	333.66	1541.24	1874.90
34	1.48	14.00	3.10	235.99	1250.52	1486.51
35	1.41	14.00	2.90	267.35	1492.76	1760.11
36	1.38	14.00	2.80	259.48	1443.04	1702.52
37	1.64	14.00	3.60	288.99	1200.59	1489.58
38	1.29	14.00	2.30	331.33	1278.37	1609.69
39	1.51	14.00	3.20	257.86	1201.83	1459.70
40	1.64	14.00	3.60	283.26	1191.24	1474.50
41	1.26	14.00	2.20	303.57	1533.43	1837.00
42	1.73	14.00	3.80	195.97	739.28	935.25
43	1.26	14.00	2.20	258.73	1421.11	1679.84
44	1.44	14.00	3.00	329.85	1548.18	1878.03
45	1.32	14.00	2.40	342.06	1462.74	1804.80

46	1.44	14.00	3.00	334.80	1508.12	1842.92
47	1.43	14.00	2.70	280.35	1183.74	1464.09
48	1.47	14.00	3.10	340.39	1494.90	1835.28
49	1.46	14.00	3.10	304.57	1312.61	1617.19
50	1.69	14.00	3.70	199.44	940.55	1139.99
51	1.64	14.00	3.60	280.98	1168.77	1449.75
52	1.52	14.00	3.30	341.47	1403.35	1744.83
53	1.41	14.00	2.90	254.30	1460.80	1715.11
54	1.25	14.00	2.00	352.72	1318.67	1671.39
55	1.51	14.00	3.20	238.67	1079.43	1318.10
56	1.44	14.00	3.00	329.99	1356.57	1686.57
57	1.29	14.00	2.30	333.46	1480.18	1813.64
58	1.47	14.00	3.10	336.68	1492.01	1828.69
59	1.41	14.00	2.90	309.87	1361.03	1670.90
60	1.38	14.00	2.80	298.04	1366.97	1665.01
61	1.41	14.00	2.90	267.13	1480.91	1748.03
62	1.40	14.00	2.90	291.41	1200.51	1491.92
63	1.41	14.00	2.90	260.20	1491.40	1751.60
64	1.26	14.00	2.20	312.96	1346.16	1659.12
65	1.29	14.00	2.30	330.69	1508.49	1839.17
66	1.47	14.00	3.10	342.46	1501.42	1843.88
67	1.43	14.00	3.00	301.38	1182.60	1483.98
68	1.44	14.00	3.00	327.74	1479.68	1807.42
69	1.41	14.00	2.90	325.82	1356.72	1682.54
70	1.41	14.00	2.90	303.12	1561.21	1864.33
71	1.44	14.00	3.00	336.34	1537.86	1874.20
72	1.44	14.00	3.00	325.27	1553.87	1879.14
73	1.47	14.00	3.10	346.19	1514.79	1860.98
74	1.49	14.00	3.20	324.28	1427.12	1751.40
75	1.47	14.00	3.10	339.75	1489.98	1829.73
76	1.44	14.00	3.00	329.81	1554.92	1884.73
77	1.46	14.00	3.10	312.38	1201.54	1513.92
78	1.49	14.00	3.20	327.08	1425.26	1752.34
79	1.41	14.00	2.90	308.17	1557.02	1865.19
80	1.44	14.00	3.00	333.98	1498.83	1832.81
81	1.41	14.00	2.90	319.86	1361.46	1681.32
82	1.46	14.00	3.10	313.13	1342.61	1655.74
83	1.26	14.00	2.20	298.31	1543.74	1842.04
84	1.52	14.00	3.30	343.84	1389.07	1732.91
85	1.51	14.00	3.20	204.54	1142.99	1347.54
86	1.29	14.00	2.30	334.83	1322.84	1657.67
87	1.47	14.00	3.10	342.76	1475.58	1818.34
88	1.41	14.00	2.90	307.41	1545.55	1852.96
89	1.46	14.00	3.10	315.48	1362.47	1677.95
90	1.41	14.00	2.90	313.66	1367.89	1681.55
91	1.32	14.00	2.40	349.91	1487.17	1837.08
92	1.32	14.00	2.40	345.09	1478.18	1823.28
93	1.29	14.00	2.30	329.34	1497.61	1826.94
94	1.32	14.00	2.40	345.91	1489.51	1835.43
95	1.41	14.00	2.90	306.57	1490.55	1797.12

96	1.44	14.00	3.00	334.94	1498.89	1833.83
97	1.32	14.00	2.40	343.14	1462.04	1805.18
98	1.41	14.00	2.90	325.28	1313.11	1638.39
99	1.66	14.00	3.60	172.49	967.26	1139.74
100	1.25	14.00	2.00	412.51	1417.39	1829.90

Appendix S2. Expected number of cases for infected, recovery and exposed

Date	Infected	Recovery	Exposed	Date	Infected	Recovery	Exposed
22-Jan	425	28	426	7-Mar	3121	185309	605
23-Jan	537	58	532	8-Mar	2542	186089	484
24-Jan	676	97	664	9-Mar	2068	186725	388
25-Jan	849	145	829	10-Mar	1680	187242	311
26-Jan	1064	206	1034	11-Mar	1364	187662	250
27-Jan	1333	282	1290	12-Mar	1106	188003	201
28-Jan	1668	377	1607	13-Mar	896	188279	161
29-Jan	2084	496	1999	14-Mar	726	188503	130
30-Jan	2602	645	2482	15-Mar	588	188685	104
31-Jan	3243	831	3075	16-Mar	476	188832	84
1-Feb	4037	1062	3799	17-Mar	385	188951	68
2-Feb	5015	1351	4677	18-Mar	311	189047	55
3-Feb	6216	1709	5733	19-Mar	252	189125	44
4-Feb	7683	2153	6989	20-Mar	203	189188	36
5-Feb	9464	2702	8465	21-Mar	164	189238	29
6-Feb	11610	3377	10172	22-Mar	133	189280	23
7-Feb	14171	4207	12108	23-Mar	107	189313	19
8-Feb	14664	7750	17346	24-Mar	87	189340	15
9-Feb	16780	11416	22496	25-Mar	70	189361	12
10-Feb	20084	15611	27606	26-Mar	57	189379	10
11-Feb	24265	20632	32449	27-Mar	46	189393	8
12-Feb	29015	26698	36597	28-Mar	37	189404	6
13-Feb	33960	33952	39545	29-Mar	30	189414	5
14-Feb	38652	42442	40874	30-Mar	24	189421	4
15-Feb	42614	52105	40409	31-Mar	19	189427	3
16-Feb	45430	62758	38286	1-Apr	16	189432	3
17-Feb	46835	74116	34898	2-Apr	13	189436	2
18-Feb	46759	85824	30762	3-Apr	10	189439	2
19-Feb	45323	97514	26371	4-Apr	8	189442	1
20-Feb	42782	108845	22107	5-Apr	7	189444	1
21-Feb	39456	119540	18213	6-Apr	5	189445	1
22-Feb	35663	129404	14809	7-Apr	4	189447	1
23-Feb	31684	138320	11925	8-Apr	4	189448	1
24-Feb	27738	146241	9537	9-Apr	3	189449	0
25-Feb	23982	153175	7591	10-Apr	2	189449	0
26-Feb	20517	159171	6025	11-Apr	2	189450	0
27-Feb	17396	164300	4774	12-Apr	1	189451	0
28-Feb	14639	168649	3781	13-Apr	1	189451	0
29-Feb	12239	172309	2995	14-Apr	1	189451	0
1-Mar	10178	175369	2374	15-Apr	1	189451	0

2-Mar	8424	177913	1883	16-Apr	1	189452	0
3-Mar	6946	180019	1496	17-Apr	1	189452	0
4-Mar	5708	181756	1191	18-Apr	0	189452	0
5-Mar	4678	183183	949	19-Apr	0	189452	0
6-Mar	3825	184352	757	20-Apr	0	189452	0
